# Potential Opportunities and Challenges of Deploying Next Generation Sequencing and CRISPR-Cas Systems to Support Diagnostics and Surveillance Towards Malaria Control and Elimination in Africa

**DOI:** 10.3389/fcimb.2022.757844

**Published:** 2022-07-13

**Authors:** Beatus M. Lyimo, Zachary R. Popkin-Hall, David J. Giesbrecht, Celine I. Mandara, Rashid A. Madebe, Catherine Bakari, Dativa Pereus, Misago D. Seth, Ramadhan M. Ngamba, Ruth B. Mbwambo, Bronwyn MacInnis, Daniel Mbwambo, Issa Garimo, Frank Chacky, Sijenunu Aaron, Abdallah Lusasi, Fabrizio Molteni, Ritha Njau, Jane A. Cunningham, Samwel Lazaro, Ally Mohamed, Jonathan J. Juliano, Jeffrey A. Bailey, Deus S. Ishengoma

**Affiliations:** ^1^ National Institute for Medical Research, Dar es Salaam, Tanzania; ^2^ School of Life Sciences and Bio-Engineering, Nelson Mandela African Institution of Science and Technology, Arusha, Tanzania; ^3^ School of Medicine, University of North Carolina, Chapel Hill, NC, United States; ^4^ Pathology and Laboratory Medicine, Center for International Health Research, Brown University, Providence, RI, United States; ^5^ Immunology and Infectious Diseases, Harvard T.H. Chan School of Public Health, Boston, MA, United States; ^6^ Infectious Disease and Microbiome Program, Broad Institute, Boston, MA, United States; ^7^ National Malaria Control Programme, Dodoma, Tanzania; ^8^ Swiss Tropical Public Health Institute, Dar es Salaam, Tanzania; ^9^ World Health Organization, Country Office, Dar es Salaam, Tanzania; ^10^ Global Malaria Programme, World Health Organization, Headquarters, Geneva, Switzerland; ^11^ Faculty of Pharmaceutical Sciences, Monash University, Melbourne, VIC, Australia

**Keywords:** next generation sequencing, CRISPR-Cas systems, pathogen genomics, malaria diagnosis, malaria molecular surveillance, Africa, Tanzania

## Abstract

Recent developments in molecular biology and genomics have revolutionized biology and medicine mainly in the developed world. The application of next generation sequencing (NGS) and CRISPR-Cas tools is now poised to support endemic countries in the detection, monitoring and control of endemic diseases and future epidemics, as well as with emerging and re-emerging pathogens. Most low and middle income countries (LMICs) with the highest burden of infectious diseases still largely lack the capacity to generate and perform bioinformatic analysis of genomic data. These countries have also not deployed tools based on CRISPR-Cas technologies. For LMICs including Tanzania, it is critical to focus not only on the process of generation and analysis of data generated using such tools, but also on the utilization of the findings for policy and decision making. Here we discuss the promise and challenges of NGS and CRISPR-Cas in the context of malaria as Africa moves towards malaria elimination. These innovative tools are urgently needed to strengthen the current diagnostic and surveillance systems. We discuss ongoing efforts to deploy these tools for malaria detection and molecular surveillance highlighting potential opportunities presented by these innovative technologies as well as challenges in adopting them. Their deployment will also offer an opportunity to broadly build in-country capacity in pathogen genomics and bioinformatics, and to effectively engage with multiple stakeholders as well as policy makers, overcoming current workforce and infrastructure challenges. Overall, these ongoing initiatives will build the malaria molecular surveillance capacity of African researchers and their institutions, and allow them to generate genomics data and perform bioinformatics analysis in-country in order to provide critical information that will be used for real-time policy and decision-making to support malaria elimination on the continent.

## Overview

Significant progress was made between 2005 and 2015 in malaria control globally but progress has stalled in recent years (World Health Organization, 2020). Changes in malaria burden were attributed to different interventions that target both mosquito vectors and parasites, which have been scaled-up in the past two decades. Recent evidence shows an increase in malaria cases globally from 2017 to 2019 compared to previous years, but the number of deaths remained relatively unchanged from 2017 to 2019. However, in 2020, malaria deaths increased from 2019 levels, with the majority of both cases and deaths involving children and pregnant women from sub-Saharan Africa (SSA) (World Health Organization, 2021). The increase in malaria cases can be partially explained by the emergence and spread of insecticide resistance in *Anopheles* populations (Kisinza et al., 2017⁠ and antimalarial resistance in parasites (Dondorp et al., 2009; Haldar et al., 2018; Ippolito et al., 2021) both of which threaten the effectiveness of the major malaria interventions, long-lasting insecticide treated bed-nets (LLINs), indoor residual spraying (IRS) and antimalarial drugs (Tawe et al., 2018; Apinjoh et al., 2019; Morgan et al., 2020). Increased malaria cases in 2020 were also attributed to the COVID-19 pandemic, which was believed to have disrupted malaria control activities (World Health Organization, 2021). However, other potential factors such as transmission from asymptomatic reservoirs (Andolina et al., 2021, climate change, and other environmental changes caused by human activities are thought to be responsible for the resurgence of malaria (Upatham et al., 1988; Chaumeau et al., 2019; Mitchell et al., 2022).

In 2020, 29 countries in Africa contributed about 96% of all reported malaria deaths globally, despite consistent implementation of the major malaria interventions since 2002⁠ (World Health Organization, 2021). Malaria control in Africa relies mainly on the use of LLINs and effective antimalarial drugs, predominantly artemisinin-based combination therapy (ACTs) for routine case management and sulphadoxine/pyrimethamine (SP) for intermittent preventive therapy in pregnant women (iPTp) (World Health Organization, 2021). Despite the detection of insecticide resistance and occurrence of mutations in *PfKelch13* gene (associated with resistance to artemisinins) in Rwanda, Uganda and Eritrea, treatment failure rates remain below 10%, hence both ACT and vector control measures are still considered highly effective (World Health Organization, 2021). However, a robust surveillance and response strategy is urgently needed to detect any early failures in the current interventions and facilitate effective response to ensure malaria elimination by 2030.

At present, malaria case management and routine surveillance rely on traditional diagnostic methods based on microscopy and rapid diagnostic tests (RDTs). These methods are widely deployed by the National Malaria Control Programmes (NMCPs) in most malaria endemic countries in Africa (Tawe et al., 2018; Abbas et al., 2019; Tessema et al., 2019b; Morgan et al., 2020). Despite their short turnaround time, these methods have major limitations (Apinjoh et al., 2019 (**Table 1**). In areas of low transmission and those closer to malaria elimination, microscopy and RDTs are less sensitive and unable to detect transmissible but very low parasite densities (Nsanzabana, 2019; Andolina et al., 2021)⁠⁠. Since most of the widely used RDTs are based on the detection of histidine rich protein 2 (HRP2), the situation is further exacerbated by the emergence and spread of *Plasmodium falciparum* with histidine rich protein 2 and 3 (*pfhrp2* and *pfhrp3)* gene deletions (Thomson et al., 2019; Feleke et al., 2021). To strengthen the current surveillance system, innovative methods of malaria diagnosis and population surveillance based on advanced molecular techniques are urgently needed to support effective case management (Ishengoma et al., 2019⁠.

**Table 1:** Various methods used for diagnostic and surveillance of malaria.

Method	Target	Sensitivity(percentage of true positives detected)	Specificity(percentage of true negatives detected)	Limit of detection	Cost per sample (USD)	Time	Advantages	Limitations	Reference
Microscopy	N/A	95%	98%	50–200 parasites/μL of blood	$0.12–$0.40	60 min	Identification of parasite morphologies, species and stage	Requires trained personnel and microscopes	(Hopkins et al., 2013; Pham et al., 2018; Mbanefo and Kumar, 2020)
Rapid Diagnostic Test (RDT)	PfHRP2, *Pf*LDH	85% to 94.8%	95.2% to 99%	50–200 parasites/μL of blood	$0.60-$2.50	15–30 min	Fast and easy to use	Mutation in *pfhrp-2* leading to false negatives, Unable to quantify parasitaemia, can produce false-positive results well after resolution of infection	(Cordray and Richards-Kortum, 2012; Hopkins et al., 2013; Pham et al., 2018; Mbanefo and Kumar, 2020)
Polymerase Chain Reaction (PCR)	18S rRNA, *cox3*, TARE-2, *varATS* and *Pfs25*	98% to 100%	88% to 94%	0.5–5 parasites/μL of blood	$0.35–$5.00	1-2 h	Low limit of detection makes it easier to detect low parasitaemia, High throughput, detects drug-resistant parasites, mixed infections	Requires expensive instruments and reagents and is not able to quantify parasitaemia	(Tangpukdee et al., 2009; Cordray and Richards-Kortum, 2012; Hopkins et al., 2013; [Bibr B90][Bibr B70])
High-volume quantitative PCR (qPCR)	*Plasmodium* sp. 18S RNA	100%	99.75%.	0.1 parasite/µl of blood	$0.50	45 min-2h	Low limit of detection makes it easier to detect low parasitaemia	Requires expensive instruments and reagents, Requires trained personnel	(Kamau et al., 2013; Imwong et al., 2014; Haanshuus et al., 2019)
Nucleic Acid Sequence-Based Amplification (NASBA)	18S mRNA	97.4–100%	80.9–94%	0.01–0.1 parasites/μL of blood	$5-$20	1–2 h	No thermocycler needed	Requires highly trained personnel, expensive	(Cordray and Richards-Kortum, 2012; Pham et al., 2018; Mbanefo and Kumar, 2020)
Loop-mediated Isothermal Amplification (LAMP)	18S rRNA, mDNA	98.3% to 100%	94.3% to 100%	1–5 parasites/μL of blood	$0.28-$5.31	30–60 min	Low limit of detection, faster reaction time than PCR, no thermocycler needed, high throughput	Easily susceptible to contamination	(Cordray and Richards-Kortum, 2012; Pham et al., 2018; Mbanefo and Kumar, 2020)
Serological test	Detection of antibodies against parasites	69.9%	100%	50–200 parasites/μL of blood	$0.50-$5.50	30-60 min	Useful for epidemiologic surveys, Relatively cheap, Provides retrospective confirmation of malaria infection	Not suitable for the diagnosis of acute malaria, Cannot discriminate species	(She et al., 2007; Tangpukdee et al., 2009; Tusting et al., 2014; Goh et al., 2021)

cox3, Mitochondrial cytochrome c oxidase III; TARE-2,Telomere associated repetitive element; varATS, Var. gene acidic terminal sequence; PfLDH, P. falciparum lactate dehydrogenase; PfHRP2; Plasmodium falciparum histidine-rich protein 2.*Sensitivity and specificity estimates come from (Boonma et al., 2007; Han, 2013) cost estimates come from (Han, 2013) except NASBA (Mbanefo and Kumar, 2020) and serology (Tusting et al., 2014; Goh et al., 2021).

Two key technologies that are slated to have a large impact on surveillance and diagnosis of malaria are Next Generation Sequencing (NGS) and clustered regularly interspaced short palindromic repeats (CRISPR). NGS, which is also termed high-throughput or massive parallel sequencing, allows for thousands to billions of DNA or RNA fragments to be sequenced⁠ and analyzed in either a targeted or whole genome approach (Deurenberg et al., 2017; Talundzic et al., 2018). Routine malaria surveillance programs could benefit from deployment of NGS technologies, as they provide a scalable, cost-effective means of surveillance in large numbers of samples (Juliano et al., 2010; Van Tyne et al., 2011; Manske et al., 2012; Lerch et al., 2017; Ngondi et al., 2017; Tawe et al., 2018; ; Moser et al., 2021).

CRISPR and CRISPR associated protein (Cas) derived from type II CRISPR bacterial immune systems, provide the basis for a new family of assays that detect nucleic acid targets with high sensitivity and specificity. While CRISPR-Cas is mostly known to enable easy gene editing, it also has enormous diagnostic potential (Jiang et al., 2013⁠. Indeed, CRISPR-Cas systems have been utilized to detect and differentiate arbovirus strains (Gootenberg et al., 2017⁠, to diagnose SARS-CoV-2 infections (Guo et al., 2020, to distinguish pathogenic bacteria (Strich and Chertow, 2019, to diagnose cancers (Yin et al., 2019, and indeed to diagnose malaria (Lee et al., 2020; Cunningham et al., 2021).

This review discusses ongoing efforts to deploy advanced molecular tools for the diagnosis and surveillance of malaria, developing the capacity for pathogen genomics in Africa, and highlights both potential opportunities presented by these innovative technologies as well as challenges facing these efforts.

### Epidemiological Applications of NGS

**Figure 1:**
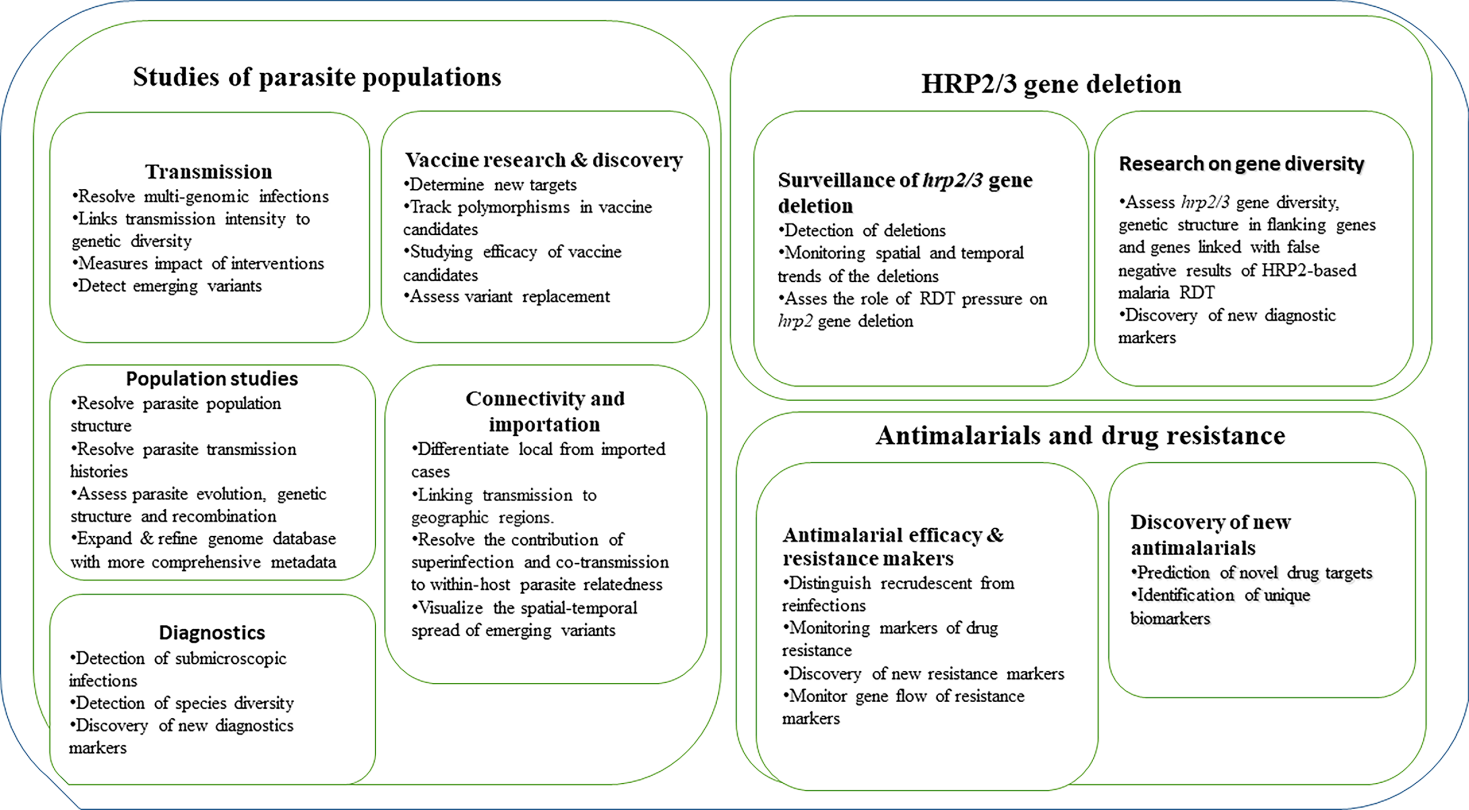
Current and potential application of NGS in studies of parasite populations, drug resistance and surveillance of hrp2/3 gene deletions.

Our ability to detect and track the spread of malaria is potentially improved by advances in malaria genomics (particularly those based on NGS) and associated bioinformatics tools in combination with epidemiological data (Escalante et al., 2015; Kwiatkowski, 2015; Watson et al., 2021)⁠. These tools can facilitate different studies of parasite populations, antimalarial drug resistance, surveillance of hrp2/3 gene deletions, and monitoring the impact of current and future interventions (**Figure 1**). Studies utilizing these methods are urgently needed and if scaled-up, can be critical for monitoring transmission to achieve proper control and the ultimate goal of malaria elimination.

#### Monitoring Malaria Transmission

Historically, transmission intensity and dynamics of malaria burden were measured using tools like entomological inoculation rates (EIR), case incidence and prevalence. These tools have helped to generate maps of mortality rates, prevalence and incidence as well as the overall global burden of malaria (Weiss et al., 2019; Neafsey et al., 2021), but recently have become less sensitive especially in areas of very low transmission. Due to their inherent limitations, they are unable to detect genetic signatures and parasite population structure indicative of modified transmission in response to interventions. For example, higher incidence could indicate a decline in the effectiveness of malaria interventions, even though the increase might actually be due to increased transmission rates from a source which was not addressed in the initial interventions (Mohd Abd Razak et al., 2016). Furthermore, decreased transmission limits outcrossing, influencing high inbreeding rates within the population and triggering population fragmentation. As such, conventional surveillance methods may result in misinterpreting the underlying processes shaping malaria epidemiology. Advances in NGS technologies supported by bioinformatic tools and increased availability of epidemiological data have made it possible to generate high quality and sensitive data which may help detect changes in transmission intensity, identify multi-genomic infections, evaluate intervention effectiveness, and identify potential deficiencies in malaria control programmes in both high and low transmission areas (Watson et al., 2021). This improved understanding can be achieved by determining the number of parasite strains, genetic diversity (e.g. within-host diversity index (*F_ws_*) as well as relatedness and genetic structure of parasites in an infection.

#### Monitoring Parasite Populations

Polymorphic genes such as merozoite surface protein (*msp1*), merozoite surface protein 2 (*msp2*) and glutamate rich protein (*glurp*) have been used extensively to study parasite genetic diversity and population structure, mostly to detect complexity/multiplicity of infection (COI/MOI), wherein multiple distinct genotypes co-occur in a single host (Lalremruata et al., 2017). Studies using these methods have demonstrated associations between MOI and such factors as host age, clinical severity, and transmission intensity (Mayengue et al., 2009; Lalremruata et al., 2017). However, these PCR-based genotyping methods have limited inter-laboratory reproducibility and also lack sensitivity, hence may underestimate allelic diversity in co-infected hosts (Assefa et al., 2014). Highly sensitive NGS tools, by contrast, can illuminate not only MOI but also parasite transmission history, genetic structure, recombination, and evolutionary history.

The use of these tools to study evolutionary history can improve our understanding of the parasite-host interactions involved in the emergence of new parasites in the region (Su et al., 2020, as well as evolutionary responses to selective pressures caused by intensified malaria elimination interventions, such as antimalarial drugs (Neafsey and Volkman, 2017). It is even possible to estimate recombination events that may lead to evasion of control and detection efforts (Miles et al., 2016). In addition, NGS data facilitates population genetic and evolutionary metric calculations, such as linkage disequilibrium (LD), effective population size (*N_e_*), site frequency spectrum (SFS), identity by descent (IBD), identity by state (IBS), haplotype diversity (H), principal component analysis (PCA), F_WS_, and F_ST_. The extent of parasite genomic data now available provides opportunities to expand and refine the global genome database and public resources, which in turn will greatly expand our understanding of parasite biology and accelerate the discovery of new interventions.

#### Parasite Connectivity and Importation

Traditionally, case incidence was used in combination with human migration data to link parasite populations across geographical and transmission gradients, which would inform intervention strategies. However, this method lacks the precision of NGS-based methods.

Genetic relatedness inferred from genomic data provides much higher geographic resolution of transmission (Tessema et al., 2019a) and can be quantified between 0 (completely unrelated parasites) and 1 (identical clones). Genetic relatedness between parasite samples is positively correlated with reduced transmission, and therefore can be used as a proxy measure of transmission level (Daniels et al., 2015; Cerqueira et al., 2017). Using advanced NGS tools, it is also possible to infer malaria transmission networks over a given timescale in a population (Ajibola et al., 2021). Importation of malaria parasites from higher to lower transmission areas, particularly those in the elimination phase, remains a major challenge to malaria elimination, and is difficult to detect using traditional methods. However, NGS methods are able to do so, as was demonstrated in Zanzibar (Morgan et al., 2020). Strengthening malaria molecular surveillance (MMS) using advanced genomic tools will reveal the number of secondary cases arising from each imported infection, allowing NMCPs to determine what interventions are required to prevent re-establishment of transmission. The data derived from effective MMS systems will also help to resolve the contribution of superinfection and co-transmission (Nair et al., 2014; Nkhoma et al., 2018).

### Using NGS to Address Challenges in Malaria Control and Elimination

#### Antimalarials and Drug Resistance

Antimalarial resistance is a major obstacle to the effective control and elimination of malaria⁠. Malaria parasites have evolved resistance to all widely deployed antimalarial drugs, leading to withdrawal of some of the drugs such as chloroquine and SP (Ishengoma et al., 2019). Antimalarial drug resistance has spread globally, although it remains rare and localized in some areas with lower transmission and therefore lower selective pressure (Haldar et al., 2018). We can clearly use these methods to retrospectively understand how and why resistance emerged and spread. However, their prospective use by NMCPs through enhancing genomic surveillance infrastructure can identify signatures of emerging drug resistance both when resistance mutations are known and unknown (Neafsey et al., 2021.

Antimalarial efficacy monitoring is usually done in therapeutic efficacy studies (TES) which enroll infected patients and genotype those with recurrent infection after treatment to distinguish between recrudescence (drug failure) and reinfection (World Health Organization, 2009). Traditional methods rely on the polymorphic markers mentioned above (World Health Organization, 2007) or microsatellites (Greenhouse et al., 2006; Malvy et al., 2018). While these methods are widespread, they are limited by their low throughput, poor resolution, and technical challenges regarding analysis, scoring and interpretation of results. Specifically, these methods under-estimate MOI or misclassify infections as either recrudescent or reinfection (Shaukat et al., 2012). However, new NGS methods using amplicon sequencing (Rao et al., 2016 or SNP barcodes are being optimized and may potentially replace the current methods, which would improve estimates of antimalarial efficacy especially in low transmission areas with limited diversity and polymorphisms in parasite populations.

NGS provides a robust, high throughput tool for identifying and tracking molecular markers of drug resistance (Ishengoma et al., 2019). Currently available markers can be used for spatial and temporal monitoring of resistance to different antimalarials, including both current drugs and those which were used in the past (Rao et al., 2016; Levitt et al., 2017; Nag et al., 2017; Talundzic et al., 2018). This monitoring is especially critical in the context of partial artemisinin resistance, which has already been reported in South-East Asia (SEA) (Leang et al., 2015; Takala-Harrison and Laufer, 2015). Artemisinin resistant parasites may be introduced from SEA (as drug resistance genes are known to spread between populations) (Roper et al., 2004; Dwivedi et al., 2016) or could potentially emerge independently in SSA, underscoring the necessity of this continuous surveillance.

It is important to understand the origin and flow of resistance genes in order to anticipate the diffusion of antimalarial resistance and interrupt its spread (Dwivedi et al., 2016). Current methods for doing this are expensive and less robust. For example, Sanger sequencing is considered to be useful in identification of major alleles but has low accuracy in identifying alleles with frequency less than 50% (minor alleles) (Rohlin et al., 2009). NGS based methods are promising as they are high throughput, less labor intensive, have high sensitivity in identifying all variants, even those with minor allele frequencies, and facilitate the discovery of novel markers (Tessema et al, 2019a).

#### Surveillance of *pfhrp2/3* Gene Deletions

Malaria rapid diagnostic tests (mRDTs) based on lateral flow detection of *P. falciparum* Histidine-Rich Protein 2 (*Pf*HRP2) have supported prompt and effective identification of *P*. *falciparum* malaria infections, particularly in resource poor regions. However, recent studies in various countries have reported some cases where pfHRP2 based mRDTs failed to detect malaria parasites in patients with positive results by either microscopy or PCR (Gamboa et al., 2010; Thomson et al., 2019; Bakari et al., 2020). While other factors may be involved (Murray et al., 2008, deletion of the *pfhrp2* gene (as well as the related *pfhrp3* that cross-reacts in RDTs) has also been reported to contribute to false-negative test results (Luchavez et al., 2011; Cheng et al., 2014; Thomson et al., 2019), which has significantly compromised the performance of *Pf*HRP2-based RDTs. Thus, WHO currently recommends for all malaria endemic countries to undertake surveys to identify the prevalence and trends of the deletion in space and time (World Health Organization, 2019).

NGS data enables both the detection of emergence and monitoring of *pfhrp2*/3 gene deletion frequencies in all settings (low and high transmission) in relation to the pressure caused by the use of HRP2-based RDTs. With intensified surveillance, it will therefore be possible to identify areas which may need to change diagnostic strategies for effective malaria control. The deletion is currently detected based on initial clinical evidence (e.g. a microscopy-positive result for *P.falciparum* but negative RDT) and confirmed with molecular approaches such as PCR and antigen detection assays (Cheng et al., 2014). However, some samples can be RDT-negative but have intact *pfhrp2/3* genes (Nsobya et al., 2021). Indeed, a study from the Democratic Republic of Congo that performed WGS on samples with suspected deletions showed that the genes were actually intact (Parr et al., 2021). However, recent data from the Horn of Africa, which utilized complementary molecular, immunological and sequencing assays, demonstrates that there have been multiple independent deletions in *pfhrp3*, which have been accompanied by strong selection for a single *pfhrp2* deletion due to pressure from RDTs (Feleke et al., 2021). As such, the MalariaGEN network has highlighted the utility of widely deployed NGS as a critical tool to detect the true *pfhrp2/3* gene deletion prevalence (MalariaGEN et al., 2021.

Gene diversity can potentially explain the variability in the sensitivity of *Pf*HRP2-based RDTs (Baker et al., 2005; ; Wurtz et al., 2013; Li et al., 2015; Gendrot et al., 2019). Indeed, studies conducted in different geographical settings have shown that gene diversity in the *pfhrp*2 gene plays a role in the sensitivity of RDTs especially among infections with low parasitaemia (≥ 200 asexual parasites/µl) (Baker et al., 2010). Given that countries such as Eritrea (Berhane et al., 2018 and Ethiopia (Golassa et al., 2020 have reported abnormally high prevalence of these deletions, NGS methods will likely be necessary for further characterization and monitoring of the diversity in these genes.

### NGS and CRISPR as Novel Surveillance and Diagnostic Tools

Timely and accurate diagnosis is a key element of effective malaria management. Traditional microscopy and RDT-based diagnostic methods involve detection of malaria parasites or products e.g. antigens in the blood of infected patients (Kasetsirikul et al., 2016). In other laboratories, malaria diagnosis involves molecular methods such as PCR (**Table 1**). Both microscopy and RDTs have been deployed as diagnostic tools in many malaria endemic areas, despite low sensitivity due to their high detection limits (≥ 50 parasites/μL) and limited ability to detect non-falciparum species. These deficiencies cause under-reporting of infections, specifically in areas with low transmission and for individuals with asymptomatic malaria or low density infections (Slater et al., 2019). By contrast, PCR has high sensitivity and specificity which make it capable of detecting low parasite density of less than 5 parasites/μl. This increased sensitivity will improve our ability to diagnose very low density infections, and detect asymptomatic individuals who can unknowingly contribute to disease transmission. Furthermore, the NGS-enabled ability to scan hundreds of *Plasmodium* genomes to better understand parasite biology and for novel diagnostic markers facilitates the development of tools that are species-specific, highly sensitive and agreeable to PCR or other nucleic acid amplification techniques (Lucchi et al., 2013.

In addition to the shortcomings outlined above, currently widespread robust malaria diagnostics require substantial financial investment and equipment, along with extensive personnel training. CRISPR-based diagnostics are an exciting development, as they have similar sensitivity and specificity to PCR assays, but require fewer resources, and are fast, cost-effective, and easy to use (Kostyusheva et al., 2021). The simplicity of the CRISPR-Cas based detection system allows the rapid development of diagnostic methods of a wide array of infectious diseases (Jiang et al., 2013; Gootenberg et al., 2017; Kostyusheva et al. 2021). Four CRISPR-based diagnostic methods have particular potential to replace PCR-based methods in low-resource settings: DETECTR, SHERLOCK, CARMEN, and CRISPR-Chip (Kaminski et al., 2021; Kostyusheva et al., 2021). These methods work reliably in isothermal conditions (negating the need for an expensive thermocycler) and results can be determined by lateral flow strips at the point of care, or by fluorescence read by a fluorimeter, or in some cases, with the naked eye. The use of lateral flow strips or visual inspection of fluorescence avoids the use of more technically challenging gel electrophoresis or quantification using a qPCR machine (Kaminski et al., 2021; Kostyusheva et al., 2021). CRISPR-based methods that have the potential to be developed for malaria diagnosis are described in **Table 2**.

**Table 2:** CRISPR-based diagnostics used in the context of malaria.

End Point	Method (Cas enzyme, amplification)	Advantages	Limitations	Reference
Lateral Flow	SHERLOCK Version 2 (Cas13 RPA)	Similar sensitivity to RT-PCR methods but without the need for expensive thermocyclers; highly specific species delineation capabilities; can perform drug-resistance genotyping; potential for use in mosquitoes as well as in clinical samples	Not yet ready for wide-scale field use; requires higher crRNA concentrations than SHERLOCK in other pathogens; “one-pot” approach not yet achievable; assay design costs are high	(Gootenberg et al., 2018; Kellner et al., 2019)
	SHINE Cas13 RPA)	Single step tool with high sensitivity compared to RT-qPCR. Detects virus from unextracted samples. Reduced contamination risk as amplification reaction tubes remain sealed.	Not yet validated with field samples	(Arizti-Sanz et al., 2020
	STOPCovid (Cas12 LAMP)	Sensitivity of this tool is similar to RT-qPCR Appropriate for low-complexity clinical laboratories.	Not yet validated with field samples	(Joung et al., 2020
Fluorescence/ Colorimetry	SHERLOCK VERSION 1 (Cas9, RPA)	Similar sensitivity to RT-PCR methods but without the need for expensive thermocyclers; highly specific species delineation capabilities; can perform drug-resistance genotyping; potential for use in mosquitoes as well as in clinical samples	Not yet ready for wide-scale field use; requires higher crRNA concentrations than SHERLOCK in other pathogens; “one-pot” approach not yet achievable; assay design costs are high	(Cunningham et al., 2021
	SHERLOCK version 2 (Cas13/RPA)	Multiplexable, portable, rapid, and quantitative detection platform of nucleic acids.	Not yet validated with field samples	(Gootenberg et al., 2018; Kellner et al., 2019)
	CARMEN (Cas13, PCR/RPA)	Detects all human-associated viruses with high sensitivity. Enables comprehensive subtyping of some viruses e.g. influenza A strains and multiplexed identification of dozens of HIV drug-resistance mutations	Not yet validated with field samples	(Ackerman et al., 2020
	NASBACC (Cas9/NASBA)	Can discriminate between viral strains with single base resolution	Long turnaround time Not yet validated with field samples Challenge in the sample preparation	(Pardee et al., 2016
	DETECTR	Higher sensitivity than SHERLOCK; functions in a single tube (“one-pot” approach); isothermal like SHERLOCK; all reaction components can be lyophilized (no need for refrigeration)	Not yet validated with field samples	(Lee et al., 2020

The deployment of CRISPR-based diagnostic methods for *P. falciparum* could revolutionize the field of malaria surveillance in Africa. The ability of this method to detect as little as one copy of target DNA per reaction will play a major role in the detection of both symptomatic and asymptomatic infections and any associated resistance mutations (Kwiatkowski, 2015⁠. CRISPR has already been used to detect malaria strain variants in mixed infections, an important challenge for malaria control and elimination efforts and a modality that could be extended to many areas of epidemiology (Kwiatkowski, 2015⁠. One-pot systems that are suitable for field deployment due to their minimal technological and logistical prerequisites, high diagnostic reliability, and rapid turnaround time have been developed for SARS-CoV-2 and show additional promise for the expansion of these tools in malaria-endemic countries (Nguyen et al., 2021). The development of isothermal reactions performed with lyophilized reagents and with straightforward visualization at the point-of-care are particularly revolutionary as a means to quickly, cheaply, and accurately diagnose malaria infections in low-resource and/or remote locations (Cunningham et al., 2021). While these tools are still in early development, their accuracy, ease-of-use, cost-effectiveness, and rapidity give them great potential to complement currently available tools.

## Application of NGS and CRISPR-Cas Systems in Africa: Current Status and Future Perspectives

In Africa, few studies have been undertaken to determine the diversity and other features of parasite populations using NGS methods. Previous studies utilized PCR genotyping of polymorphic genes (*msp1*, *msp2* and *glurp*) to determine parasite diversity with little focus on population structures and other population genetics metrics (Carlsson et al., 2011; Yavo et al., 2016; Metoh et al., 2020; Amoah et al., 2021). Recently, some regional studies have been conducted in Africa using genomic tools and have provided initial data on parasite population structure (Manske et al., 2012; Amambua-Ngwa et al., 2019; Abera et al., 2021; Moser et al., 2021). However, other areas, particularly those with low transmission, remain largely unexplored (Morgan et al., 2020). In addition, the metrics that are routinely used in these studies [e.g. *F*_WS_, *F*_ST_ (the fixation index), COI/MOI, IBD, inbreeding coefficient (F), and coefficient of uniqueness (COU)] have not been fully evaluated to be reliably informative for monitoring intervention-modulated changes in malaria transmission (Abera et al., 2021; Onyango et al., 2021). Therefore, further intensive study and metric development is required. The integration of traditional epidemiological data with NGS data will likely improve malaria surveillance in service of elimination strategies. While CRISPR-based diagnostics could revolutionize the field of malaria surveillance in malaria endemic countries in Africa, they have not yet been deployed or tailored to the specific needs of NMCPs.

Meanwhile, there are ongoing collaborative projects between African researchers, NMCPs, and partners in the USA and Europe to establish local molecular, genetic, and genomic laboratory and analytic capacity to support MMS. These projects are located in several countries in SSA including Senegal, Mozambique, Tanzania, Mali, The Gambia, Ghana, Gabon, Madagascar, Cameroon, Ivory Coast, Burkina Faso, Ethiopia and Uganda (Amambua-Ngwa et al., 2019; MalariaGEN et al., 2021). In Tanzania, previous efforts were made with the support of different funding agencies and partners and set-up the stage for the current initiatives (Amambua-Ngwa et al., 2019; MalariaGEN et al., 2021). Our current collaboration (Molecular Surveillance of Malaria in Tanzania, MSMT) is funded by the Bill and Melinda Gates Foundation and focuses on six key aims to attain the bold and ambitious goal of establishing local capacity for in-country generation and analysis of genomics data to support policy and decision making by the NMCP and its stakeholders. To that end, Tanzanian stakeholders identified three scientific aims for MSMT: 1) unraveling parasite population genetics, 2) country-wide drug resistance monitoring, and 3) country-wide surveys of *pfhrp2/3* gene deletion. In addition, the project will address three supportive aims with a focus on training for capacity building, career development, and science leadership for sustainability of MMS. The project will also focus on developing a reliable model for effective engagement with NMCP and other stakeholders, and developing use cases for MMS in Tanzania. Lastly, the project will develop and implement a framework and platforms for storage, management, analysis, and sharing of the genomic data and associated metadata.To successfully adopt and transfer NGS and bioinformatics tools to Africa and build local capacity, a robust strategy of capacity building is critical. The MSMT team has developed a “counterpart model” of capacity building. This model involves pairing Tanzanian researchers with US-based expert (counterparts) collaborators with the role ensuring successful technology transfer.

To ensure key local stakeholders are effectively engaged, the MMS projects should bring on board all local stakeholders and ensure they are fully engaged in the project. In Tanzania, the MSMT project established an advisory committee which is under the Chief Medical Officer of the Tanzanian Ministry of Health with the role of providing oversight and guiding the implementation and sustainability of the project. The project is run by the joint implementation committee, which includes the study team, NIMR, NMCP and the Ministry in charge of the local governments (which owns and runs all health facilities in Tanzania). The technical implementation committee involves NIMR researchers, NMCP and collaborating partners who are advised by a committee of experts with experience and track records in MMS and capacity building particularly in Africa.

Thus far, a genomics laboratory has been established at NIMR in Dar es Salaam and will soon be equipped with state-of-the-art equipment to ensure genomics data are generated in-country and analysed by local experts from the second year of the project in 2022. The Tanzanian researchers have sequenced over 8,500 field collected samples in laboratories in the US as part of their training, and future analysis will take place in the NIMR laboratory with further training and coaching from the US-based collaborators. It is anticipated that our training and capacity building model and the laboratory capacity to be built in Tanzania will pave a way to a sustainable MMS for supporting not only malaria but other pathogens as part of the broader pathogen genomics initiative.

## Challenges Associated With NGS Application in Africa

African researchers seeking to answer biological questions using NGS face many challenges, ranging from funding to procurement to lack of trained personnel (**Table 3**). The long-standing imbalance between collaborators in the global North and South has resulted in unsustainable patterns of research practice that must be broken if African researchers are to be free to initiate and execute NGS-dependent research studies. For the wide application of NGS technology in malaria surveillance, African countries need substantial investment to establish genomic capacity (Haanshuus et al., 2019 including human capacity, ancillary equipment for library preparation, quality control and sequencing (Escalante et al., 2015.

**Table 3:** Challenges and opportunities for NGS application in a resource constrained country such as Tanzania.

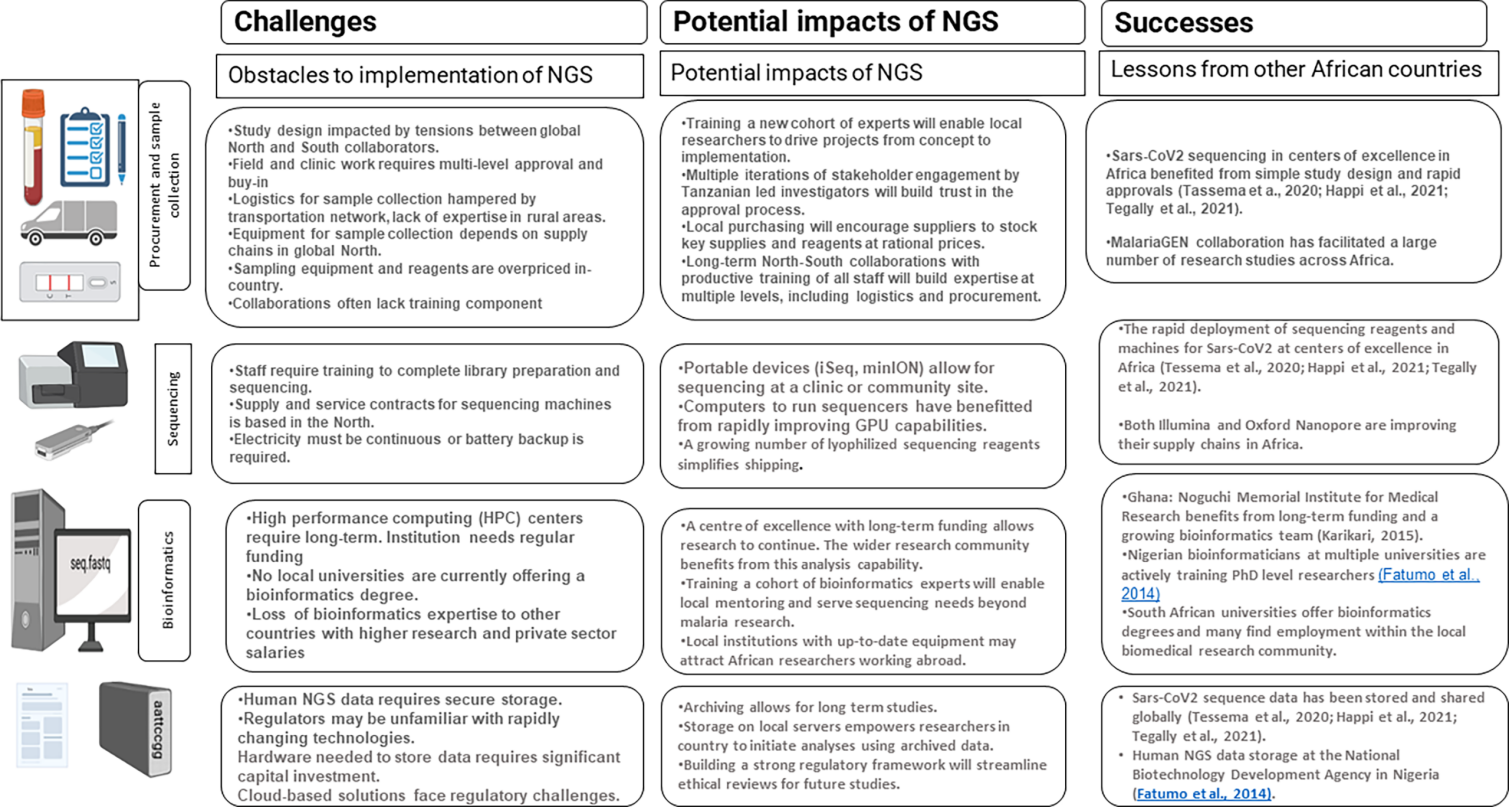	

## Study Design and Sample Collection

Historical imbalances are visible beginning with the funding and design of research programs. Too often, minimal intellectual input is sought from African researchers during this phase, and donors must critique their funding calls to address this imbalance. For large-scale studies where samples are collected from many districts within a country, buy-in from a large number of authorities is needed. The need to meet funding deadlines for an overseas collaborator may create tension during this phase. African procurement services and scientific suppliers to furnish basic sample collection necessities are often limited or unavailable, creating further imbalance between collaborators. To overcome this challenge, long-term partnerships that emphasize training are needed. Funding for NGS projects must be balanced, and local supply chains must be developed to support sample collection and sequencing without reliance on procurement abroad. One of the best recent examples of the rapid implementation of NGS for infectious disease research was seen in the centres of excellence for SARS-CoV-2 sequencing (Tessema et al., 2020; Happi et al., 2021; Tegally et al., 2021). To monitor the emergence of novel viral strains, several African laboratories rapidly pivoted to sequencing, taking advantage of publicly shared protocols, using kits and consumables tailored to this problem, and laboratories in Botswana and South Africa were able to quickly detect and raise awareness of the omicron variant of concern (Viana et al., 2022).

## Sequencing

The sequencing phase of an NGS study faces many related challenges. Until sequencing becomes routine, skilled staff are difficult to find and retain. Sequencing machines, consumables and quality control reagents are challenging to procure, and are priced above the budgets of many African laboratories. Both Illumina and Oxford Nanopore Technologies have taken steps to address procurement challenges by supplying flow cells and consumables directly to several African agencies, but gaps in procurement continue to be filled by collaborating Northern institutions. Equipment and flow cells were rapidly distributed to several laboratories early in the spread of SARS-CoV-2. Robust supply chains and investment from the major suppliers of sequencing technology are needed to expand similar supply chains.

## Bioinformatics and Data Storage Infrastructure

NGS studies produce large amounts of data that is analyzed with an ever-evolving suite of bioinformatics tools. Adequate data storage is a significant cost and requires in-house expertise. Few African institutions are training bioinformaticians, and those that are trained may leave for better paying jobs in the North-centered biotechnology industry. Building high performance computing clusters will require sustained investment, as will building up a cohort of bioinformaticians to work on African infectious disease sequencing projects. There is also a need to develop local expertise for equipment installation and maintenance to prevent long delays and additional costs accrued from overseas outsourcing (Apinjoh et al., 2019). Examples of institutions that have successfully built bioinformatics systems include the Noguchi institute in Ghana (Karikari, 2015, Redeemer’s University in Nigeria (Fatumo et al., 2014, and several South African universities.

## Long-Term Data Storage and Regulatory Frameworks

Human and pathogen sequence data is sensitive, requiring adequate long-term storage, and a regulatory framework to protect patients’ information. Relatively low-cost storage exists in cloud storage systems, but privacy concerns must be addressed for this to be allowed by regulators. Hardware for long-term storage must be budgeted for in research proposals, and efficient storage formats are needed.

**Skilled Workforce**

Despite their potential, the initial investments in infrastructure for NGS are high, the methods require highly skilled personnel to perform sample processing, bioinformaticians, molecular epidemiologists for data analysis and interpretation, field epidemiologists, disease specialists for interpretation of the generated data, and public health specialists for adoption of the findings into policy (Escalante et al., 2015; Ishengoma et al., 2019). However, there are limited initiatives to train, retain, mentor and maintain highly skilled experts in Africa. The ongoing capacity building initiatives such as those under the African Academy of Sciences (https://www.aasciences.africa/), The human heredity and Health (H3Africa Initiative, https://h3africa.org/) and others need to be strongly supported by African countries for sustainability.

## Conclusion

The application of advanced NGS tools and CRISPR-Cas technologies to malaria diagnosis and surveillance has great potential for malaria elimination in Africa. These tools will improve our understanding of malaria transmission dynamics, including our ability to track the spread of parasites and their associated clinically relevant mutations. With the wide scale distribution of genomics facilities and expertise, as well as state-of-the-art CRISPR-based diagnostics that substantially reduce equipment and training needs, our understanding of many aspects of malaria biology will dramatically expand. In addition to wider reaching and more accurate mobile and point-of-care diagnostics, African capacity for malaria control and ultimately elimination will result from this technological expansion. Furthermore, the deployment of these technologies and associated spread of expertise is not limited to malaria, but can also facilitate the better understanding and control of other infectious diseases as part of the ongoing initiative of building the capacity for pathogen genomics in Africa.

Nevertheless, significant challenges remain, which need to be addressed to facilitate smooth implementation of these tools necessary for malaria surveillance and elimination. Simulation studies which consider surveillance tools as an intervention strategy can also be performed to quantitatively evaluate deployment of these tools. Available simulation models such as openmalaria can be used to optimize these tools in terms of cost-effectiveness and public health. These challenges range from technological hurdles to a lack of funding investment and sufficiently skilled workforce in the areas where malaria interventions are most desperately needed. However, should these hurdles be overcome, the potential public health implications of deploying NGS and CRISPR-Cas tools in Tanzania and elsewhere are invaluable.

## Authors Contributions

DI- Formulated the original idea, supervised and edited the manuscript; BL, ZPH, DG, CB, DP, MS, JJ, JB, and DI - wrote and edited the manuscript. All authors contributed to the article and approved the submitted version.

## Funding

This work was supported, in whole, by the Bill & Melinda Gates Foundation [grant number 02202]. Under the grant conditions of the Foundation, a Creative Commons Attribution 4.0 Generic License has already been assigned to the Author Accepted Manuscript version that might arise from this submission. JJ also received funding from NIH K24AI134990.

## Conflict of Interest

The authors declare that the research was conducted in the absence of any commercial or financial relationships that could be construed as a potential conflict of interest.

The reviewer DN declared a shared affiliation with the author BM to the handling editor at the time of review.

## Publisher’s Note

All claims expressed in this article are solely those of the authors and do not necessarily represent those of their affiliated organizations, or those of the publisher, the editors and the reviewers. Any product that may be evaluated in this article, or claim that may be made by its manufacturer, is not guaranteed or endorsed by the publisher.

## References

[B1] Abbas N. Saba T. Rehman A. Mehmood Z. Javaid N. Tahir M.. (2019). *Plasmodium* Species Aware Based Quantification of Malaria Parasitemia in Light Microscopy Thin Blood Smear. Microsc. Res. Tech. 82, 1198–1214. doi: 10.1002/jemt.23269 30937990

[B2] Abera D. Kibet C. K. Degefa T. Amenga-Etego L. Bargul J. L. Golassa L. (2021). Genomic Analysis Reveals Independent Evolution of *Plasmodium Falciparum* Populations in Ethiopia. Malar. J. 20, 129. doi: 10.1186/s12936-021-03660-y 33663492 PMC7934276

[B3] Ackerman C. M. Myhrvold C. Thakku S. G. Freije C. A. Metsky H. C. Yang D. K.. (2020). Massively Multiplexed Nucleic Acid Detection With Cas13. Nature 582, 277–282. doi: 10.1038/s41586-020-2279-8 32349121 PMC7332423

[B4] Ajibola O. Diop M. F. Ghansah A. Amenga-Etego L. Golassa L. Apinjoh T.. (2021). In Silico Characterisation of Putative *Plasmodium Falciparum* Vaccine Candidates in African Malaria Populations. Sci. Rep. 11, 16215. doi: 10.1038/s41598-021-95442-4 34376744 PMC8355234

[B5] Amambua-Ngwa A. Amenga-Etego L. Kamau E. Amato R. Ghansah A. Golassa L.. (2019). Major Subpopulations of *Plasmodium Falciparum* in Sub-Saharan Africa. Science 365, 813–816. doi: 10.1126/science.aav5427 31439796

[B6] Amoah L. E. Abukari Z. Dawson-Amoah M. E. Dieng C. C. Lo E. Afrane Y. A. (2021). Population Structure and Diversity of *Plasmodium Falciparum* in Children With Asymptomatic Malaria Living in Different Ecological Zones of Ghana. BMC Infect. Dis. 21, 439. doi: 10.1186/s12879-021-06120-9 33985447 PMC8120845

[B7] Andolina C. Rek J. C. Briggs J. Okoth J. Musiime A. Ramjith J.. (2021). Sources of Persistent Malaria Transmission in a Setting With Effective Malaria Control in Eastern Uganda: A Longitudinal, Observational Cohort Study. Lancet Infect. Dis. 21, 1568–1578. doi: 10.1016/S1473-3099(21)00072-4 34146476 PMC8554388

[B8] Apinjoh T. O. Ouattara A. Titanji V. P. K. Djimde A. Amambua-Ngwa A. (2019). Genetic Diversity and Drug Resistance Surveillance of *Plasmodium Falciparum* for Malaria Elimination: Is There an Ideal Tool for Resource-Limited Sub-Saharan Africa? Malar. J. 18, 217. doi: 10.1186/s12936-019-2844-5 31242921 PMC6595576

[B9] Arizti-Sanz J. Freije C. A. Stanton A. C. Petros B. A. Boehm C. K. Siddiqui S.. (2020). Streamlined Inactivation, Amplification, and Cas13-Based Detection of SARS-CoV-2. Nat. Commun. 11, 5921. doi: 10.1038/s41467-020-19097-x 33219225 PMC7680145

[B10] Assefa S. A. Preston M. D. Campino S. Ocholla H. Sutherland C. J. Clark T. G. (2014). estMOI: Estimating Multiplicity of Infection Using Parasite Deep Sequencing Data. Bioinformatics 30, 1292–1294. doi: 10.1093/bioinformatics/btu005 24443379 PMC3998131

[B11] Bakari C. Jones S. Subramaniam G. Mandara C. I. Chiduo M. G. Rumisha S.. (2020). Community-Based Surveys for *Plasmodium Falciparum Pfhrp2* and *Pfhrp3* Gene Deletions in Selected Regions of Mainland Tanzania. Malar. J. 19, 391. doi: 10.1186/s12936-020-03459-3 33148255 PMC7640459

[B12] Baker J. Ho M.-F. Pelecanos A. Gatton M. Chen N. Abdullah S.. (2010). Global Sequence Variation in the Histidine-Rich Proteins 2 and 3 of *Plasmodium Falciparum*: Implications for the Performance of Malaria Rapid Diagnostic Tests. Malar. J. 9, 129. doi: 10.1186/1475-2875-9-129 20470441 PMC2893195

[B13] Baker J. McCarthy J. Gatton M. Kyle D. E. Belizario V. Luchavez J.. (2005). Genetic Diversity of *Plasmodium Falciparum* Histidine-Rich Protein 2 (PfHRP2) and its Effect on the Performance of PfHRP2-Based Rapid Diagnostic Tests. J. Infect. Dis. 192, 870–877. doi: 10.1086/432010 16088837

[B14] Berhane A. Anderson K. Mihreteab S. Gresty K. Rogier E. Mohamed S.. (2018). Major Threat to Malaria Control Programs by *Plasmodium Falciparum* Lacking Histidine-Rich Protein 2, Eritrea. Emerging Infect. Dis. 24, 462–470. doi: 10.3201/eid2403.171723 PMC5823352 29460730

[B15] Boonma P. Christensen P. R. Suwanarusk R. Price R. N. Russell B. Lek-Uthai U. (2007). Comparison of Three Molecular Methods for the Detection and Speciation of *Plasmodium Vivax* and *Plasmodium Falciparum* . Malar. J. 6, 124. doi: 10.1186/1475-2875-6-124 17868467 PMC2020467

[B16] Carlsson A. M. Ngasala B. E. Dahlström S. Membi C. Veiga I. M. Rombo L.. (2011). *Plasmodium Falciparum* Population Dynamics During the Early Phase of Anti-Malarial Drug Treatment in Tanzanian Children With Acute Uncomplicated Malaria. Malar. J. 10, 380. doi: 10.1186/1475-2875-10-380 22185672 PMC3280947

[B17] Cerqueira G. C. Cheeseman I. H. Schaffner S. F. Nair S. McDew-White M. Phyo A. P.. (2017). Longitudinal Genomic Surveillance of *Plasmodium Falciparum* Malaria Parasites Reveals Complex Genomic Architecture of Emerging Artemisinin Resistance. Genome Biol. 18, 78. doi: 10.1186/s13059-017-1204-4 28454557 PMC5410087

[B18] Chaumeau V. Kajeechiwa L. Fustec B. Landier J. Naw Nyo S. Nay Hsel S.. (2019). Contribution of Asymptomatic *Plasmodium* Infections to the Transmission of Malaria in Kayin State, Myanmar. J. Infect. Dis. 219, 1499–1509. doi: 10.1093/infdis/jiy686 30500927 PMC6467188

[B19] Cheng Q. Gatton M. L. Barnwell J. Chiodini P. McCarthy J. Bell D.. (2014). *Plasmodium Falciparum* Parasites Lacking Histidine-Rich Protein 2 and 3: A Review and Recommendations for Accurate Reporting. Malar. J. 13, 283. doi: 10.1186/1475-2875-13-283 25052298 PMC4115471

[B20] Cordray M. S. Richards-Kortum R. R. (2012). Emerging Nucleic Acid-Based Tests for Point-of-Care Detection of Malaria. Am. J. Trop. Med. Hyg. 87, 223–230. doi: 10.4269/ajtmh.2012.11-0685 22855751 PMC3414556

[B21] Cunningham C. H. Hennelly C. M. Lin J. T. Ubalee R. Boyce R. M. Mulogo E. M.. (2021). A Novel CRISPR-Based Malaria Diagnostic Capable of *Plasmodium* Detection, Species Differentiation, and Drug-Resistance Genotyping. EBioMedicine 68, 103415. doi: 10.1016/j.ebiom.2021.103415 34139428 PMC8213918

[B22] Daniels R. F. Schaffner S. F. Wenger E. A. Proctor J. L. Chang H.-H. Wong W.. (2015). Modeling Malaria Genomics Reveals Transmission Decline and Rebound in Senegal. Proc. Natl. Acad. Sci. U.S.A. 112, 7067–7072. doi: 10.1073/pnas.1505691112 25941365 PMC4460456

[B23] Deurenberg R. H. Bathoorn E. Chlebowicz M. A. Couto N. Ferdous M. García-Cobos S.. (2017). Application of Next Generation Sequencing in Clinical Microbiology and Infection Prevention. J. Biotechnol. 243, 16–24. doi: 10.1016/j.jbiotec.2016.12.022 28042011

[B24] Dondorp A. M. Nosten F. Yi P. Das D. Phyo A. P. Tarning J.. (2009). Artemisinin Resistance in *Plasmodium Falciparum* Malaria. N. Engl. J. Med. 361, 455–467. doi: 10.1056/NEJMoa0808859 19641202 PMC3495232

[B25] Dwivedi A. Khim N. Reynes C. Ravel P. Ma L. Tichit M.. (2016). *Plasmodium Falciparum* Parasite Population Structure and Gene Flow Associated to Anti-Malarial Drugs Resistance in Cambodia. Malar. J. 15, 319. doi: 10.1186/s12936-016-1370-y 27301553 PMC4908689

[B26] Escalante A. A. Ferreira M. U. Vinetz J. M. Volkman S. K. Cui L. Gamboa D.. (2015). Malaria Molecular Epidemiology: Lessons From the International Centers of Excellence for Malaria Research Network. Am. J. Trop. Med. Hyg. 93, 79–86. doi: 10.4269/ajtmh.15-0005 26259945 PMC4574277

[B27] Fatumo S. A. Adoga M. P. Ojo O. O. Oluwagbemi O. Adeoye T. Ewejobi I.. (2014). Computational Biology and Bioinformatics in Nigeria. PloS Comput. Biol. 10, e1003516. doi: 10.1371/journal.pcbi.1003516 24763310 PMC3998874

[B28] Feleke S. M. Reichert E. N. Mohammed H. Brhane B. G. Mekete K. Mamo H.. (2021). *Plasmodium Falciparum* is Evolving to Escape Malaria Rapid Diagnostic Tests in Ethiopia. Nat. Microbiol. 6, 1289–1299. doi: 10.1038/s41564-021-00962-4 34580442 PMC8478644

[B29] Gamboa D. Ho M.-F. Bendezu J. Torres K. Chiodini P. L. Barnwell J. W.. (2010). A Large Proportion of *P. Falciparum* Isolates in the Amazon Region of Peru Lack *Pfhrp2* and *Pfhrp3*: Implications for Malaria Rapid Diagnostic Tests. PloS One 5, e8091. doi: 10.1371/journal.pone.0008091 20111602 PMC2810332

[B30] Gendrot M. Fawaz R. Dormoi J. Madamet M. Pradines B. (2019). Genetic Diversity and Deletion of *Plasmodium Falciparum* Histidine-Rich Protein 2 and 3: A Threat to Diagnosis of *P. Falciparum* Malaria. Clin. Microbiol. Infect. 25, 580–585. doi: 10.1016/j.cmi.2018.09.009 30267926

[B31] Goh B. Ching K. Soares Magalhães R. J. Ciocchetta S. Edstein M. D. Maciel-de-Freitas R.. (2021). The Application of Spectroscopy Techniques for Diagnosis of Malaria Parasites and Arboviruses and Surveillance of Mosquito Vectors: A Systematic Review and Critical Appraisal of Evidence. PloS Negl. Trop. Dis. 15, e0009218. doi: 10.1371/journal.pntd.0009218 33886567 PMC8061870

[B32] Golassa L. Messele A. Amambua-Ngwa A. Swedberg G. (2020). High Prevalence and Extended Deletions in *Plasmodium Falciparum Hrp2/3* Genomic Loci in Ethiopia. PloS One 15, e0241807. doi: 10.1371/journal.pone.0241807 33152025 PMC7644029

[B33] Gootenberg J. S. Abudayyeh O. O. Kellner M. J. Joung J. Collins J. J. Zhang F. (2018). Multiplexed and Portable Nucleic Acid Detection Platform With Cas13, Cas12a, and Csm6. Science 360, 439–444. doi: 10.1126/science.aaq0179 29449508 PMC5961727

[B34] Gootenberg J. S. Abudayyeh O. O. Lee J. W. Essletzbichler P. Dy A. J. Joung J.. (2017). Nucleic Acid Detection With CRISPR-Cas13a/C2c2. Science 356, 438–442. doi: 10.1126/science.aam9321 28408723 PMC5526198

[B35] Greenhouse B. Myrick A. Dokomajilar C. Woo J. M. Carlson E. J. Rosenthal P. J.. (2006). Validation of Microsatellite Markers for Use in Genotyping Polyclonal *Plasmodium Falciparum* Infections. Am. J. Trop. Med. Hyg. 75, 836–842. doi: 10.4269/ajtmh.2006.75.836 17123974 PMC1697796

[B36] Guo L. Sun X. Wang X. Liang C. Jiang H. Gao Q.. (2020). SARS-CoV-2 Detection With CRISPR Diagnostics. Cell Discovery 6, 34. doi: 10.1038/s41421-020-0174-y 32435508 PMC7235268

[B37] Haanshuus C. G. Mørch K. Blomberg B. Strøm G. E. A. Langeland N. Hanevik K.. (2019). Assessment of Malaria Real-Time PCR Methods and Application With Focus on Low-Level Parasitaemia. PloS One 14, e0218982. doi: 10.1371/journal.pone.0218982 31276473 PMC6611585

[B38] Haldar K. Bhattacharjee S. Safeukui I. (2018). Drug Resistance in *Plasmodium* . Nat. Rev. Microbiol. 16, 156–170. doi: 10.1038/nrmicro.2017.161 29355852 PMC6371404

[B39] Han E.-T. (2013). Loop-Mediated Isothermal Amplification Test for the Molecular Diagnosis of Malaria. Expert Rev. Mol. Diagn. 13, 205–218. doi: 10.1586/erm.12.144 23477559

[B40] Happi A. N. Ugwu C. A. Happi C. T. (2021). Tracking the Emergence of New SARS-CoV-2 Variants in South Africa. Nat. Med. 27, 372–373. doi: 10.1038/s41591-021-01265-1 33723453

[B41] Hopkins H. González I. J. Polley S. D. Angutoko P. Ategeka J. Asiimwe C.. (2013). Highly Sensitive Detection of Malaria Parasitemia in a Malaria-Endemic Setting: Performance of a New Loop-Mediated Isothermal Amplification Kit in a Remote Clinic in Uganda. J. Infect. Dis. 208, 645–652. doi: 10.1093/infdis/jit184 23633405 PMC3719898

[B42] Imwong M. Hanchana S. Malleret B. Rénia L. Day N. P. J. Dondorp A.. (2014). High-Throughput Ultrasensitive Molecular Techniques for Quantifying Low-Density Malaria Parasitemias. J. Clin. Microbiol. 52, 3303–3309. doi: 10.1128/JCM.01057-14 24989601 PMC4313154

[B43] Ippolito M. M. Moser K. A. Kabuya J.-B. B. Cunningham C. Juliano J. J. (2021). Antimalarial Drug Resistance and Implications for the WHO Global Technical Strategy. Curr. Epidemiol. Rep. 8, 46–62. doi: 10.1007/s40471-021-00266-5 33747712 PMC7955901

[B44] Ishengoma D. S. Saidi Q. Sibley C. H. Roper C. Alifrangis M. (2019). Deployment and Utilization of Next-Generation Sequencing of *Plasmodium Falciparum* to Guide Anti-Malarial Drug Policy Decisions in Sub-Saharan Africa: Opportunities and Challenges. Malar. J. 18, 267. doi: 10.1186/s12936-019-2853-4 31477109 PMC6719357

[B45] Jiang W. Bikard D. Cox D. Zhang F. Marraffini L. A. (2013). RNA-Guided Editing of Bacterial Genomes Using CRISPR-Cas Systems. Nat. Biotechnol. 31, 233–239. doi: 10.1038/nbt.2508 23360965 PMC3748948

[B46] Joung J. Ladha A. Saito M. Kim N.-G. Woolley A. E. Segel M.. (2020). Detection of SARS-CoV-2 With SHERLOCK One-Pot Testing. N. Engl. J. Med. 383, 1492–1494. doi: 10.1056/NEJMc2026172 32937062 PMC7510942

[B47] Juliano J. J. Ariey F. Sem R. Tangpukdee N. Krudsood S. Olson C.. (2009). Misclassification of Drug Failure in *Plasmodium Falciparum* Clinical Trials in Southeast Asia. J. Infect. Dis. 200, 624–628. doi: 10.1086/600892 19591576 PMC2761972

[B48] Juliano J. J. Porter K. Mwapasa V. Sem R. Rogers W. O. Ariey F.. (2010). Exposing Malaria in-Host Diversity and Estimating Population Diversity by Capture-Recapture Using Massively Parallel Pyrosequencing. Proc. Natl. Acad. Sci. U.S.A. 107, 20138–20143. doi: 10.1073/pnas.1007068107 21041629 PMC2993407

[B49] Kamau E. Alemayehu S. Feghali K. C. Saunders D. Ockenhouse C. F. (2013). Multiplex qPCR for Detection and Absolute Quantification of Malaria. PloS One 8, e71539. doi: 10.1371/journal.pone.0071539 24009663 PMC3756973

[B50] Kaminski M. M. Abudayyeh O. O. Gootenberg J. S. Zhang F. Collins J. J. (2021). CRISPR-Based Diagnostics. Nat. Biomed. Eng. 5, 643–656. doi: 10.1038/s41551-021-00760-7 34272525

[B51] Karikari T. K. (2015). Bioinformatics in Africa: The Rise of Ghana? PloS Comput. Biol. 11, e1004308. doi: 10.1371/journal.pcbi.1004308 26378921 PMC4574930

[B52] Kasetsirikul S. Buranapong J. Srituravanich W. Kaewthamasorn M. Pimpin A. (2016). The Development of Malaria Diagnostic Techniques: A Review of the Approaches With Focus on Dielectrophoretic and Magnetophoretic Methods. Malar. J. 15, 358. doi: 10.1186/s12936-016-1400-9 27405995 PMC4942956

[B53] Kellner M. J. Koob J. G. Gootenberg J. S. Abudayyeh O. O. Zhang F. (2019). SHERLOCK: Nucleic Acid Detection With CRISPR Nucleases. Nat. Protoc. 14, 2986–3012. doi: 10.1038/s41596-019-0210-2 31548639 PMC6956564

[B54] Kisinza W. N. Nkya T. E. Kabula B. Overgaard H. J. Massue D. J. Mageni Z.. (2017). Multiple Insecticide Resistance in *Anopheles Gambiae* From Tanzania: A Major Concern for Malaria Vector Control. Malar. J. 16, 439. doi: 10.1186/s12936-017-2087-2 29084560 PMC5663032

[B55] Kostyusheva A. Brezgin S. Babin Y. Vasilyeva I. Glebe D. Kostyushev D. (2021). CRISPR-Cas Systems for Diagnosing Infectious Diseases. Methods. 203, 431–446. doi: 10.1016/j.ymeth.2021.04.007 33839288 PMC8032595

[B56] Kwiatkowski D. (2015). Malaria Genomics: Tracking a Diverse and Evolving Parasite Population. Int. Health 7, 82–84. doi: 10.1093/inthealth/ihv007 25733556 PMC4379983

[B57] Lalremruata A. Jeyaraj S. Engleitner T. Joanny F. Lang A. Bélard S.. (2017). Species and Genotype Diversity of *Plasmodium* in Malaria Patients From Gabon Analysed by Next Generation Sequencing. Malar. J. 16, 398. doi: 10.1186/s12936-017-2044-0 28974215 PMC5627438

[B58] Leang R. Taylor W. R. J. Bouth D. M. Song L. Tarning J. Char M. C.. (2015). Evidence of *Plasmodium Falciparum* Malaria Multidrug Resistance to Artemisinin and Piperaquine in Western Cambodia: Dihydroartemisinin-Piperaquine Open-Label Multicenter Clinical Assessment. Antimicrob. Agents Chemother. 59, 4719–4726. doi: 10.1128/AAC.00835-15 26014949 PMC4505193

[B59] Lee R. A. Puig H. D. Nguyen P. Q. Angenent-Mari N. M. Donghia N. M. McGee J. P.. (2020). Ultrasensitive CRISPR-Based Diagnostic for Field-Applicable Detection of *Plasmodium* Species in Symptomatic and Asymptomatic Malaria. Proc. Natl. Acad. Sci. U.S.A. 117, 25722–25731. doi: 10.1073/pnas.2010196117 32958655 PMC7568265

[B60] Lerch A. Koepfli C. Hofmann N. E. Messerli C. Wilcox S. Kattenberg J. H.. (2017). Development of Amplicon Deep Sequencing Markers and Data Analysis Pipeline for Genotyping Multi-Clonal Malaria Infections. BMC Genomics 18, 864. doi: 10.1186/s12864-017-4260-y 29132317 PMC5682641

[B61] Levitt B. Obala A. Langdon S. Corcoran D. O’Meara W. P. Taylor S. M. (2017). Overlap Extension Barcoding for the Next Generation Sequencing and Genotyping of *Plasmodium Falciparum* in Individual Patients in Western Kenya. Sci. Rep. 7, 41108. doi: 10.1038/srep41108 28117350 PMC5259759

[B62] Li P. Xing H. Zhao Z. Yang Z. Cao Y. Li W.. (2015). Genetic Diversity of *Plasmodium Falciparum* Histidine-Rich Protein 2 in the China-Myanmar Border Area. Acta Trop. 152, 26–31. doi: 10.1016/j.actatropica.2015.08.003 26297799 PMC4918506

[B63] Lucchi N. W. Oberstaller J. Kissinger J. C. Udhayakumar V. (2013). Malaria Diagnostics and Surveillance in the Post-Genomic Era. Public Health Genomics 16, 37–43. doi: 10.1159/000345607 23548716 PMC4694569

[B64] Luchavez J. Baker J. Alcantara S. Belizario V. Cheng Q. McCarthy J. S.. (2011). Laboratory Demonstration of a Prozone-Like Effect in HRP2-Detecting Malaria Rapid Diagnostic Tests: Implications for Clinical Management. Malar. J. 10, 286. doi: 10.1186/1475-2875-10-286 21957869 PMC3214175

[B65] MalariaGEN Ahouidi A. Ali M. Almagro-Garcia J. Amambua-Ngwa A. Amaratunga C.. (2021). An Open Dataset of *Plasmodium Falciparum* Genome Variation in 7,000 Worldwide Samples. Wellcome Open Res. 6, 42. doi: 10.12688/wellcomeopenres.16168.2 33824913 PMC8008441

[B66] Malvy D. Torrentino-Madamet M. L’Ollivier C. Receveur M.-C. Jeddi F. Delhaes L.. (2018). *Plasmodium Falciparum* Recrudescence Two Years After Treatment of an Uncomplicated Infection Without Return to an Area Where Malaria Is Endemic. Antimicrob. Agents Chemother. 62, e01892-17. doi: 10.1128/AAC.01892-17 29229635 PMC5786779

[B67] Manske M. Miotto O. Campino S. Auburn S. Almagro-Garcia J. Maslen G.. (2012). Analysis of *Plasmodium Falciparum* Diversity in Natural Infections by Deep Sequencing. Nature 487, 375–379. doi: 10.1038/nature11174 22722859 PMC3738909

[B68] Matiya D. J. Philbert A. B. Kidima W. Matowo J. J. (2019). Dynamics and Monitoring of Insecticide Resistance in Malaria Vectors Across Mainland Tanzania From 1997 to 2017: A Systematic Review. Malar. J. 18, 102. doi: 10.1186/s12936-019-2738-6 30914051 PMC6434877

[B69] Mayengue P. I. Luty A. J. F. Rogier C. Baragatti M. Kremsner P. G. Ntoumi F. (2009). The Multiplicity of *Plasmodium Falciparum* Infections is Associated With Acquired Immunity to Asexual Blood Stage Antigens. Microbes Infect. 11, 108–114. doi: 10.1016/j.micinf.2008.10.012 19028595

[B70] Mbanefo A. Kumar N. (2020). Evaluation of Malaria Diagnostic Methods as a Key for Successful Control and Elimination Programs. Trop. Med. Infect. Dis. 5 (2), 102. doi: 10.3390/tropicalmed5020102 PMC7344938 32575405

[B71] Metoh T. N. Chen J.-H. Fon-Gah P. Zhou X. Moyou-Somo R. Zhou X.-N. (2020). Genetic Diversity of *Plasmodium Falciparum* and Genetic Profile in Children Affected by Uncomplicated Malaria in Cameroon. Malar. J. 19, 115. doi: 10.1186/s12936-020-03161-4 32188442 PMC7081701

[B72] Miles A. Iqbal Z. Vauterin P. Pearson R. Campino S. Theron M.. (2016). Indels, Structural Variation, and Recombination Drive Genomic Diversity in *Plasmodium Falciparum* . Genome Res. 26, 1288–1299. doi: 10.1101/gr.203711.115 27531718 PMC5052046

[B73] Mitchell C. L. Janko M. M. Mwandagalirwa M. K. Tshefu A. K. Edwards J. K. Pence B. W.. (2022). Impact of Extractive Industries on Malaria Prevalence in the Democratic Republic of the Congo: A Population-Based Cross-Sectional Study. Sci. Rep. 12, 1737. doi: 10.1038/s41598-022-05777-9 35110617 PMC8810856

[B74] Mohd Abd Razak M. R. Sastu U. R. Norahmad N. A. Abdul-Karim A. Muhammad A. Muniandy P. K.. (2016). Genetic Diversity of *Plasmodium Falciparum* Populations in Malaria Declining Areas of Sabah, East Malaysia. PloS One 11, e0152415. doi: 10.1371/journal.pone.0152415 27023787 PMC4811561

[B75] Morgan A. P. Brazeau N. F. Ngasala B. Mhamilawa L. E. Denton M. Msellem M.. (2020). Falciparum Malaria From Coastal Tanzania and Zanzibar Remains Highly Connected Despite Effective Control Efforts on the Archipelago. Malar. J. 19, 47. doi: 10.1186/s12936-020-3137-8 31992305 PMC6988337

[B76] Moser K. A. Madebe R. A. Aydemir O. Chiduo M. G. Mandara C. I. Rumisha S. F.. (2021). Describing the Current Status of *Plasmodium Falciparum* Population Structure and Drug Resistance Within Mainland Tanzania Using Molecular Inversion Probes. Mol. Ecol. 30, 100–113. doi: 10.1111/mec.15706 33107096 PMC8088766

[B77] Murray C. K. Gasser R. A. Magill A. J. Miller R. S. (2008). Update on Rapid Diagnostic Testing for Malaria. Clin. Microbiol. Rev. 21, 97–110. doi: 10.1128/CMR.00035-07 18202438 PMC2223842

[B78] Nag S. Dalgaard M. D. Kofoed P.-E. Ursing J. Crespo M. Andersen L. O.. (2017). High Throughput Resistance Profiling of *Plasmodium Falciparum* Infections Based on Custom Dual Indexing and Illumina Next Generation Sequencing-Technology. Sci. Rep. 7, 2398. doi: 10.1038/s41598-017-02724-x 28546554 PMC5445084

[B79] Nair S. Nkhoma S. C. Serre D. Zimmerman P. A. Gorena K. Daniel B. J.. (2014). Single-Cell Genomics for Dissection of Complex Malaria Infections. Genome Res. 24, 1028–1038. doi: 10.1101/gr.168286.113 24812326 PMC4032849

[B80] Neafsey D. E. Taylor A. R. MacInnis B. L. (2021). Advances and Opportunities in Malaria Population Genomics. Nat. Rev. Genet. 22, 502–517. doi: 10.1038/s41576-021-00349-5 33833443 PMC8028584

[B81] Neafsey D. E. Volkman S. K. (2017). Malaria Genomics in the Era of Eradication. Cold Spring Harb. Perspect. Med. 7, a025544. doi: 10.1101/cshperspect.a025544 28389516 PMC5538406

[B82] Ngondi J. M. Ishengoma D. S. Doctor S. M. Thwai K. L. Keeler C. Mkude S.. (2017). Surveillance for Sulfadoxine-Pyrimethamine Resistant Malaria Parasites in the Lake and Southern Zones, Tanzania, Using Pooling and Next-Generation Sequencing. Malar. J. 16, 236. doi: 10.1186/s12936-017-1886-9 28583119 PMC5460401

[B83] Nguyen L. T. Macaluso N. C. Pizzano B. L. M. Cash M. N. Spacek J. Karasek J.. (2021). A Thermostable Cas12b From *Brevibacillus* Leverages One-Pot Detection of SARS-CoV-2 Variants of Concern. eBioMedicine, 77,103926. doi: 10.1016/j.ebiom.2022.103926 PMC8917962 35290826

[B84] Nkhoma S. C. Banda R. L. Khoswe S. Dzoole-Mwale T. J. Ward S. A. (2018). Intra-Host Dynamics of Co-Infecting Parasite Genotypes in Asymptomatic Malaria Patients. Infect. Genet. Evol. 65, 414–424. doi: 10.1016/j.meegid.2018.08.018 30145390 PMC6219893

[B85] Nsanzabana C. (2019). Strengthening Surveillance Systems for Malaria Elimination by Integrating Molecular and Genomic Data. Trop. Med. Infect. Dis. 4 (4), 139. doi: 10.3390/tropicalmed4040139 PMC6958499 31816974

[B86] Nsobya S. L. Walakira A. Namirembe E. Kiggundu M. Nankabirwa J. I. Ruhamyankaka E.. (2021). Deletions of *Pfhrp2* and *Pfhrp3* Genes Were Uncommon in Rapid Diagnostic Test-Negative *Plasmodium Falciparum* Isolates From Uganda. Malar. J. 20, 4. doi: 10.1186/s12936-020-03547-4 33386076 PMC7777526

[B87] Onyango S. A. Ochwedo K. O. Machani M. G. Omondi C. J. Debrah I. Ogolla S. O.. (2021). Genetic Diversity and Population Structure of the Human Malaria Parasite *Plasmodium Falciparum* Surface Protein Pfs47 in Isolates From the Lowlands in Western Kenya. PloS One 16, e0260434. doi: 10.1371/journal.pone.0260434 34843560 PMC8629314

[B88] Pardee K. Green A. A. Takahashi M. K. Braff D. Lambert G. Lee J. W.. (2016). Rapid, Low-Cost Detection of Zika Virus Using Programmable Biomolecular Components. Cell 165, 1255–1266. doi: 10.1016/j.cell.2016.04.059 27160350

[B89] Parr J. B. Kieto E. Phanzu F. Mansiangi P. Mwandagalirwa K. Mvuama N.. (2021). Analysis of False-Negative Rapid Diagnostic Tests for Symptomatic Malaria in the Democratic Republic of the Congo. Sci. Rep. 11, 6495. doi: 10.1038/s41598-021-85913-z 33753817 PMC7985209

[B90] Pham N. M. Karlen W. Beck H.-P. Delamarche E. (2018). Malaria and the “Last” Parasite: How can Technology Help? Malar. J. 17, 260. doi: 10.1186/s12936-018-2408-0 29996831 PMC6042346

[B91] Rao P. N. Uplekar S. Kayal S. Mallick P. K. Bandyopadhyay N. Kale S.. (2016). A Method for Amplicon Deep Sequencing of Drug Resistance Genes in *Plasmodium Falciparum* Clinical Isolates From India. J. Clin. Microbiol. 54, 1500–1511. doi: 10.1128/JCM.00235-16 27008882 PMC4879288

[B92] Rohlin A. Wernersson J. Engwall Y. Wiklund L. Björk J. Nordling M. (2009). Parallel Sequencing Used in Detection of Mosaic Mutations: Comparison With Four Diagnostic DNA Screening Techniques. Hum. Mutat. 30, 1012–1020. doi: 10.1002/humu.20980 19347965

[B93] Roper C. Pearce R. Nair S. Sharp B. Nosten F. Anderson T. (2004). Intercontinental Spread of Pyrimethamine-Resistant Malaria. Science 305 (5687), 1124. doi: 10.1126/science.1098876 15326348

[B94] Shaukat A. M. Gilliams E. A. Kenefic L. J. Laurens M. B. Dzinjalamala F. K. Nyirenda O. M.. (2012). Clinical Manifestations of New Versus Recrudescent Malaria Infections Following Anti-Malarial Drug Treatment. Malar. J. 11, 207. doi: 10.1186/1475-2875-11-207 22709627 PMC3489583

[B95] She R. C. Rawlins M. L. Mohl R. Perkins S. L. Hill H. R. Litwin C. M. (2007). Comparison of Immunofluorescence Antibody Testing and Two Enzyme Immunoassays in the Serologic Diagnosis of Malaria. J. Travel Med. 14, 105–111. doi: 10.1111/j.1708-8305.2006.00087.x 17367480

[B96] Slater H. C. Ross A. Felger I. Hofmann N. E. Robinson L. Cook J.. (2019). The Temporal Dynamics and Infectiousness of Subpatent *Plasmodium Falciparum* Infections in Relation to Parasite Density. Nat. Commun. 10, 1433. doi: 10.1038/s41467-019-09441-1 30926893 PMC6440965

[B97] Strich J. R. Chertow D. S. (2019). CRISPR-Cas Biology and Its Application to Infectious Diseases. J. Clin. Microbiol. 57, e01307-18. doi: 10.1128/JCM.01307-18 30429256 PMC6440769

[B98] Su X.-Z. Zhang C. Joy D. A. (2020). Host-Malaria Parasite Interactions and Impacts on Mutual Evolution. Front. Cell. Infect. Microbiol. 10. doi: 10.3389/fcimb.2020.587933 PMC7652737 33194831

[B99] Takala-Harrison S. Laufer M. K. (2015). Antimalarial Drug Resistance in Africa: Key Lessons for the Future. Ann. N. Y. Acad. Sci. 1342, 62–67. doi: 10.1111/nyas.12766 25891142 PMC4527866

[B100] Talundzic E. Ravishankar S. Kelley J. Patel D. Plucinski M. Schmedes S.. (2018). Next-Generation Sequencing and Bioinformatics Protocol for Malaria Drug Resistance Marker Surveillance. Antimicrob. Agents Chemother. 62, e02474-17. doi: 10.1128/AAC.02474-17 29439965 PMC5913988

[B101] Tangpukdee N. Duangdee C. Wilairatana P. Krudsood S. (2009). Malaria Diagnosis: A Brief Review. Korean J. Parasitol. 47, 93–102. doi: 10.3347/kjp.2009.47.2.93 19488414 PMC2688806

[B102] Tawe L. Menegon M. Ramatlho P. Muthoga C. W. Mutukwa N. Vurayai M.. (2018). Molecular Surveillance of *Plasmodium Falciparum* Drug Resistance Markers in Clinical Samples From Botswana. Am. J. Trop. Med. Hyg. 99, 1499–1503. doi: 10.4269/ajtmh.18-0440 30350774 PMC6283485

[B103] Tegally H. Wilkinson E. Lessells R. J. Giandhari J. Pillay S. Msomi N.. (2021). Sixteen Novel Lineages of SARS-CoV-2 in South Africa. Nat. Med. 27, 440–446. doi: 10.1038/s41591-021-01255-3 33531709

[B104] Tessema S. K. Inzaule S. C. Christoffels A. Kebede Y. de Oliveira T. Ouma A. E. O.. (2020). Accelerating Genomics-Based Surveillance for COVID-19 Response in Africa. Lancet Microbe 1, e227–e228. doi: 10.1016/S2666-5247(20)30117-8 32838350 PMC7434434

[B105] Tessema S. K. Raman J. Duffy C. W. Ishengoma D. S. Amambua-Ngwa A. Greenhouse B. (2019b). Applying Next-Generation Sequencing to Track *Falciparum* Malaria in Sub-Saharan Africa. Malar. J. 18, 268. doi: 10.1186/s12936-019-2880-1 31477139 PMC6720407

[B106] Tessema S. Wesolowski A. Chen A. Murphy M. Wilheim J. Mupiri A.-R.. (2019a). Using Parasite Genetic and Human Mobility Data to Infer Local and Cross-Border Malaria Connectivity in Southern Africa. eLife 8, e43510. doi: 10.7554/eLife.43510 30938286 PMC6478435

[B107] Thomson R. Beshir K. B. Cunningham J. Baiden F. Bharmal J. Bruxvoort K. J.. (2019). *Pfhrp2* and *Pfhrp3* Gene Deletions That Affect Malaria Rapid Diagnostic Tests for *Plasmodium Falciparum*: Analysis of Archived Blood Samples From 3 African Countries. J. Infect. Dis. 220, 1444–1452. doi: 10.1093/infdis/jiz335 31249999 PMC6761929

[B108] Tusting L. S. Bousema T. Smith D. L. Drakeley C. (2014). Measuring Changes in *Plasmodium Falciparum* Transmission: Precision, Accuracy and Costs of Metrics. Adv. Parasitol. 84, 151–208. doi: 10.1016/B978-0-12-800099-1.00003-X 24480314 PMC4847140

[B109] Upatham E. S. Prasittisuk C. Ratanatham S. Green C. A. Rojanasunan W. Setakana P.. (1988). Bionomics of *Anopheles Maculatus* Complex and Their Role in Malaria Transmission in Thailand. Southeast Asian J. Trop. Med. Public Health 19, 259–269. 3227404

[B110] Van Tyne D. Park D. J. Schaffner S. F. Neafsey D. E. Angelino E. Cortese J. F.. (2011). Identification and Functional Validation of the Novel Antimalarial Resistance Locus *PF10_0355* in *Plasmodium Falciparum* . PloS Genet. 7, e1001383. doi: 10.1371/journal.pgen.1001383 21533027 PMC3080868

[B111] Viana R. Moyo S. Amoako D. G. Tegally H. Scheepers C. Althaus C. L.. (2022). Rapid Epidemic Expansion of the SARS-CoV-2 Omicron Variant in Southern Africa. Nature 603, 679–686. doi: 10.1038/s41586-022-04411-y 35042229 PMC8942855

[B112] Watson O. J. Okell L. C. Hellewell J. Slater H. C. Unwin H. J. T. Omedo I.. (2021). Evaluating the Performance of Malaria Genetics for Inferring Changes in Transmission Intensity Using Transmission Modeling. Mol. Biol. Evol. 38, 274–289. doi: 10.1093/molbev/msaa225 32898225 PMC7783189

[B113] Weiss D. J. Lucas T. C. D. Nguyen M. Nandi A. K. Bisanzio D. Battle K. E.. (2019). Mapping the Global Prevalence, Incidence, and Mortality of Plasmodium Falciparum 2000-17: A Spatial and Temporal Modelling Study. Lancet. 394 (10195), 322–331. doi: 10.1016/S0140-6736(19)31097-9 31229234 PMC6675740

[B114] World Health Organization (2007) Methods and Techniques for Clinical Trials on Antimalarial Drug Efficacy: Genotyping to Identify Parasite Populations (World Health Organization). Available at: http://www.who.int/malaria/publications/atoz/9789241596305/en/ (Accessed October 8, 2020).

[B115] World Health Organization (2009). Methods for Surveillance of Antimalarial Drug Efficacy (Geneva: World Health Organization).

[B116] World Health Organization (2019). Response Plan to Pfhrp2 Gene Deletions (World Health Organization).

[B117] World Health Organization (2020). Master Protocol for Surveillance of Pfhrp2/3 Deletions and Biobanking to Support Future Research (World Health Organization).

[B118] World Health Organization (2021). World Malaria Report 2021 (Geneva: World Health Organization).

[B119] Wurtz N. Fall B. Bui K. Pascual A. Fall M. Camara C.. (2013). *Pfhrp2* and *Pfhrp3* Polymorphisms in *Plasmodium Falciparum* Isolates From Dakar, Senegal: Impact on Rapid Malaria Diagnostic Tests. Malar. J. 12, 34. doi: 10.1186/1475-2875-12-34 23347727 PMC3571878

[B120] Yavo W. Konaté A. Mawili-Mboumba D. P. Kassi F. K. Tshibola Mbuyi M. L. Angora E. K.. (2016). Genetic Polymorphism of *Msp1* and *Msp2* in *Plasmodium Falciparum* Isolates From Côte D’Ivoire Versus Gabon. J. Parasitol. Res. 2016, 3074803. doi: 10.1155/2016/3074803 27110390 PMC4823507

[B121] Yin H. Xue W. Anderson D. G. (2019). CRISPR-Cas: A Tool for Cancer Research and Therapeutics. Nat. Rev. Clin. Oncol. 16, 281–295. doi: 10.1038/s41571-019-0166-8 30664678

